# Further Researches on the Pathology of Phlegmasia Dolens

**Published:** 1853-09

**Authors:** Robert Lee


*Further Researches on the Pathology of Phlegmasia Dolens.* By ROBERT LEE, M D., F.R.S. At a late meeting of the Royal Medical and Chirurgical Society of London, Dr. Lee read a paper on this subject. The author commenced his paper by observing that it was not till the publication of the memoirs of M. Bouillaud, M. Velpeau, and the late Dr. Davis, that the true nature of this disease was known. Up to this period various hypotheses had been advanced respecting the cause of the swelling in the lower extremities of puerperal women—mere speculations unsupported by facts; but the cases and directions of the authors just enumerated demonstrated that the true nature of the disease consisted in an inflammation of the trunks and principal branches of the veins of the lower extremities. In papers by the author, published in the fifteenth volume of the Transactions, the actual condition of the iliac and femoral veins was ascertained, and he has been led to infer that inflammation of the veins gave rise to all the phenomena in puerperal women of phlegmasia dolens, and that it commenced in the uterine branches of the hypogastric veins, and subsequently extended from them into the iliac and femoral trunks of the affected side. Other cases had been recorded in the Transactions, of crural phlebitis following ulceration of the mucous membrane of the intestines. Experiments performed by Pirigott, in 1839, and by Reumert, in 1840, on dogs, showed that the action of chemical and mechanical irritants was limited to the vein on which the experiment was made, and the extension of the inflammation in the veins was not common; and Stanius, who had collated and tested all the facts bearing on the subject, doubted whether inflammation of venous trunks admitted of being excited by constitutional causes, independently of local irritation. A series of experiments on the veins of the lower animals, similar to those just mentioned, had recently been made and a paper on phlegmasia dolens had been read to the Society during the present session, not founded on actual observation of the disease as it occurs in the human subject, but upon experiments on the veins of the lower animals in which phlegmasia dolens had never been observed. The object of the present communication was to submit to the Society the observations which the author had made during the last twenty-four years in inflammation of the crural veins. The paper contained the record of forty-three cases of phlegmasia dolens. The first nine cases were accompanied by post mortem descriptions, and preparations illustrating the disease; and the author was led, from the whole of the facts thus adduced, to the conclusions he had formerly expressed, "that inflammation of the iliac and femoral veins gave rise to all the phenomena of phlegmasia dolens, and that the inflammation commenced in the uterine branches of the hypogastric veins, and from them extended to the iliac and femoral trunks of the affected side." The next series comprised the history of twenty cases, which the author thought furnished additional evidence in favor of this conclusion, though, in consequence of the recovery of the greater number of the patients, an opportunity was not afforded of determining by dissection the actual condition of the crural veins. Nine cases followed, which demonstrated that phlegmasia dolens might occur wholly unconnected with pregnancy and parturition, and that in such cases the inflammation likewise commenced in the uterine branches of the hypogastric veins, and followed a course similar to what occurred in puerperal cases. In some of these the inflammation of the uterine veins was produced by cancerous disease of the os and cervix uteri; in others there was no organic diseases of any kind previously existing. The concluding cases were five, in which crural phlebitis had followed inflammation of the saphena veins, and of the deep veins of the lower extremities from fra ture of the tibia and fibula, and the pressure of encephaloid tumors on the thoracic viscera. The author thought that these cases and dissections, as well as those of the distinguished authors whom he had quoted, proved in the most conclusive manner that inflammation of the iliac and femoral veins was the proximate cause of phlegmasia dolens, and that in puerperal women this inflammation commenced in the uterine branches of the hypogastric veins. It had likewise been demonstrated by morbid anatomy that phlegmasia dolens was a disease which might take place in women who had never been pregnant, and even in the male sex, and that, under all circumstances, the approximate cause was the same.

Dr. Mayo inquired if, in the cases of phlegmasia dolens recorded by Dr. Lee, any peculiarity antecedent to the inflammation of the veins had been noticed?

Mr. Streeter asked if Dr. Lee had statistical information to give respecting the comparative frequency of the disease?

Dr. John Clark inquired if phlegmasia dolens, in the common acceptation of the term, and independent of other disorders, was always the result of inflammation of the veins. He thought the disease generally was very mild, and, in the experience of writers on the subject, not a fatal one.

Dr. Mackenzie said, that in the discussion of such questions as that which is now before the society, it appeared to be important to distinguish between the facts which are alleged and the conclusions which are drawn from them. Now, in the present case, the facts alleged are that certain lesions of the crural veins are developed in the progress of phlegmasia dolens. The conclusions are that such lesions constitute the essence or proximate cause of the disease. He (Dr. Mackenzie) assented fully and entirely to the first of these propositions, whilst he dissented as fully and entirely from the latter; and as he was unwilling to enter upon the discussion of this question in a controversial spirit, he would make no reference whatever to the investigation which he had lately submitted to the Society on the subject of this disease, but would confine himself to a statement of such facts as were known to the profession, which appeared to him to be opposed to the theory of the disease which had been affirmed by Dr. Lee. The disease known as phlegmasia dolens was a very complex malady. It was one which was characterized not only by a morbid condition of the veins, but by a morbid condition of the sensory, the motor, the lymphatic, and the secretory organs of the affected extremity also; and, accordingly, in all well-marked cases of the disease there was exquisite sensibility of the limb, especially in the track of particular nerves, loss of motor power, amounting sometimes to perfect immobility of the extremity, inflammation and obstruction of the lymphatic vessels and glands, and a general hot, tense, and elastic swelling of the limb, not simply arising from œdema, but possessing rather the character of active exudation than of passive effusion. Now, could all these lesions depend upon or be deduced from mere inflammation and obstruction of the principal vein of the extremity? Were they ordinarily observed in cases of simple uncomplicated phlebitis? Or, if not, was there any thing in the anatomical or physiological characters of the veins to justify our deducing *à priori* from it? And if we replied to these questions, as he submitted that we must, in the negative, he should ask whether those who adopt this theory have undertaken any particular investigation for the purpose of determining this point? Or, in other words, have they reproduced the lesion of the veins in a simple, uncomplicated form, and observed such consequences to follow. Now, to these questions we must also reply in the negative, and it must be added that the whole matter rested purely on assumption. It had been assumed that because the crural veins were found obviously diseased in fatal cases of phlegmasia dolens, such lesions constituted the proximate cause of the disease. No further steps had been taken to establish the truth of this doctrine, and that, therefore, had been taken as a matter of assumption which ought to have been made a matter of demonstration. Further, he would observe that the clinical history of the disease and the progress of symptoms did not support this theory. It was quite true that in some cases the first irritations commenced in the region of the femoral vessels, but in others it was far otherwise; in some they commenced in the back, in others in the hip, sometimes in the calf of the leg, and more frequently in the popliteal region. Again, one leg might be affected alone, or both concurrently; or the disease, after having attacked one, may pass on to the other, or a superior extremity might be affected; and he had lately met with a case in which, after symptoms of the disease had successively declared themselves in the left lower and upper extremities, the malady ultimately established itself in the right arm, the whole right upper extremity being hot, swollen, and tense, the surface exquisitely painful, with loss of motor power, and a tense, corded condition of the basilic vein. Now, it appeared to him that these facts were inconsistent with the theory that the proximate cause of the disease was essentially inflammation of the crural veins. They pointed to the existence of some more general and diffusive cause, in regard to which it was probable that phlebitis itself was but a secondary affection. Again, he would point to the general experience of the profession as being opposed to this theory. It was now upwards of thirty years since it was first promulgated by his friend and teacher, the late Dr. David Davis; and although the facts upon which it rested were well known, it was yet very far from being generally adopted. Thus, in this country, Dr. Burns affirmed that the nerves were as much affected as the veins. Others regarded the lymphatic vessels as being principally affected; while many, dissatisfied with these restricted views of the pathology of the disease, preferred the theory of the late Dr. Hull, that it consisted in a general inflammation of the several organs and structures of the affected limb. So again, on the continent, the greatest difference of opinion existed respecting its nature and pathology; and whilst many affirmed that it consisted essentially in inflammation of the lymphatics, and others that it was a specific inflammation of the cellular tissue, nearly all agreed that in its general characters it differed widely from ordinary phlebitis. Now, this diversity of opinion existed notwithstanding that all were aware of the facts upon which the phlebitic theory of the disease rested, and it afforded a powerful argument against it, because it tended to show that when tested by general experience, and considered irrespectively of particular facts, and free from bias, it failed to account rationally for all the known phenomena of the disease, and consequently could not be regarded as its proximate cause. Then in the sequela of the disease, circumstances are met with which are inconsistent with this theory. We know, for instance, that after an attack of the disease, the crural veins were generally left impervious or obliterated, and yet it would happen that successive attacks of the disease might occur in the same extremity. Now, if it was true that the first attack left them in the condition described, it was difficult to understand how, having functionally ceased to exist, they could again take on functional activity, and become the seat of active inflammation. So also it happened after an attack of the disease, that the limb would be left for many years, or even for the remainder of life, in a weak, sensitive, and irritable condition, being easily affected by atmospheric and constitutional influences. It was easy to reconcile these facts with the notion that the nerves had been injured or damaged by the attack, but not with the idea that the veins alone had been affected. On all these grounds, then, it appeared to him (Dr. Mackenzie) that the phlebitic theory of the disease was either defective or erronious. But assuming for a moment that it was correct, he would yet observe that it left much which was still to be explained. We had yet to learn the nature of that peculiar inflammation of the veins which was so exceptional and so different from ordinary phlebitis. Did it depend upon some peculiar dispositiou on the part of the venous coats to take on diffusive inflammation, or did it depend primarily upon the blood? If we adopted the first of these theories, we were bound to state the nature of the peculiarity, and the laws of its development. For to be satisfied with merely giving it a name, and to speak of it as a "specific" inflammation, was not to advance our scientific knowledge, but rather to take refuge, or to hide our ignorance under the shadow of a name. If, on the other hand, we accepted the latter view, and regarded the venous inflammation as dependent upon some morbid condition of blood, then, indeed, we might reasonably account not only for the peculiarities it presented, but for all the several lesions of other organs, and the structural changes with which it was associated. Upon this view, also, we might reconcile the conflicting opinions respecting the nature of the disease which had been held by different pathologists, and the variations which it manifested in its symptoms and progress in different cases. But in accepting this view we must forego the theory that the phlebitis was the proximate cause of the disease, and regard it, as it really was, as a secondary rather than a primary phenomenon; related to the other lesions of the extremity not so much in the order of cause and effect, but as being like them a parallel effect of some more general and diffusive morbific agent.

Dr. Lee, in his reply, entered into the literary history of the disease under discussion, from the time of Mauriceau to the present. He particularly dwelt upon the facts originally published by the late Dr. Davis, and showed at great length his (Dr. Lee's) own labors in this disease. In answer to Dr. Mayo, he said that there were no antecedent symptoms calling for any treatment. He knew nothing of the comparative frequency of the disease. In respect to the views advanced by Dr. Mackenzie, he could only say that whilst he (Dr. Lee) admitted that there might be a blood disease present, it was consecutive to the inflammation in the veins. That inflammation was in reality the proximate cause of the disease. The scalpel had shown this; he thought that was sufficient, and could not understand what further was required.—*London Lancet.*

